# Supplementary figures and images for: RNA‐seq Analysis of Peri‐Implant Tissue Shows Differences in Immune, Notch, Wnt, and Angiogenesis Pathways in Aged Versus Young Mice
Source: JBMR Plus. 2021 Sep 9;5(11):e10535. doi: 10.1002/jbm4.10535 (PMC8567488; doi:10.1002/jbm4.10535)

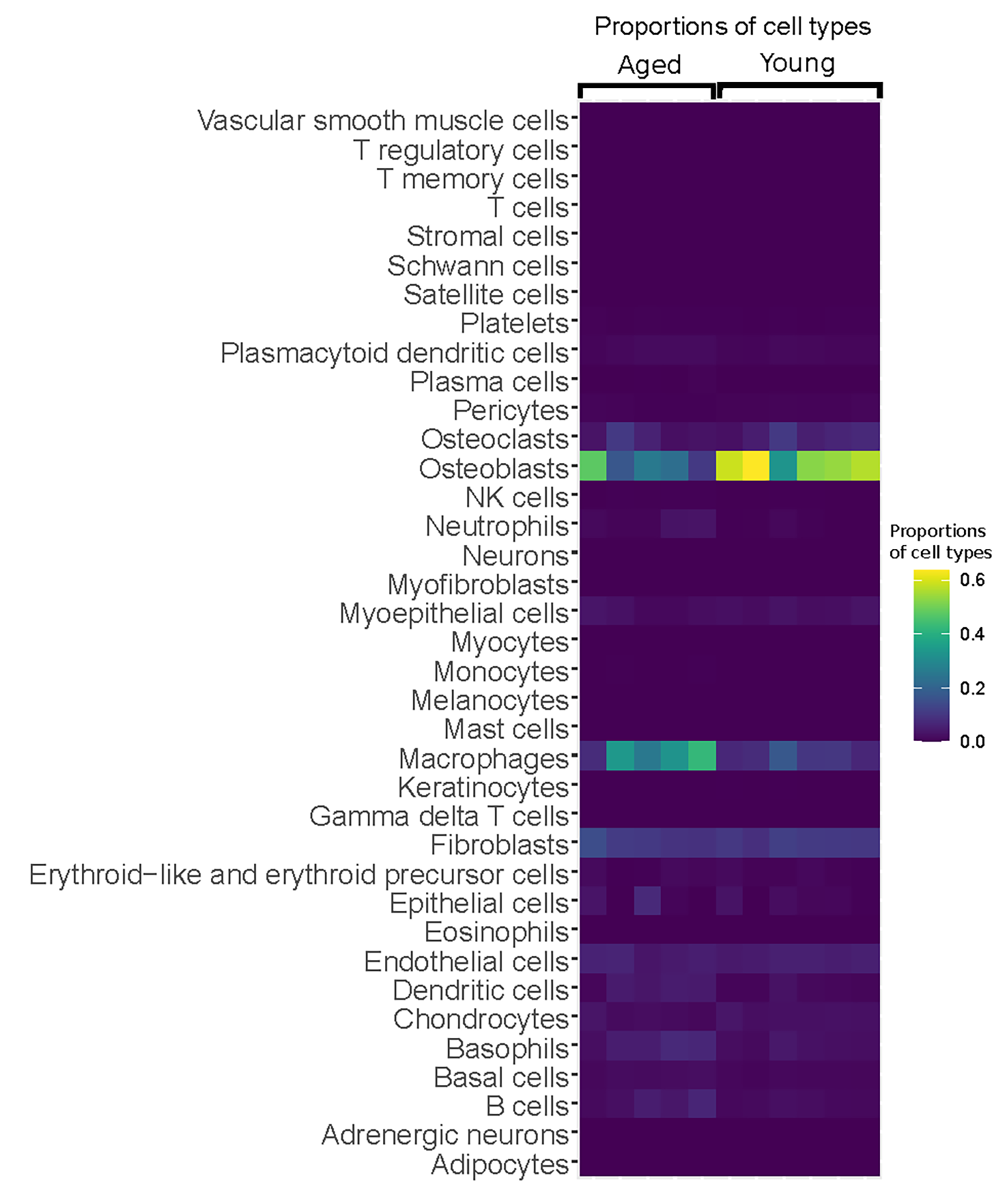

Supplement: Supplementary file 1 — Supplemental Fig. S1. Average proportion of cell types from the BICS for each replica from the young and aged animals. The proportion of cell types has been inferred using SCDC algorithm as described in Materials and Methods. [file JBM4-5-e10535-s006.tif]

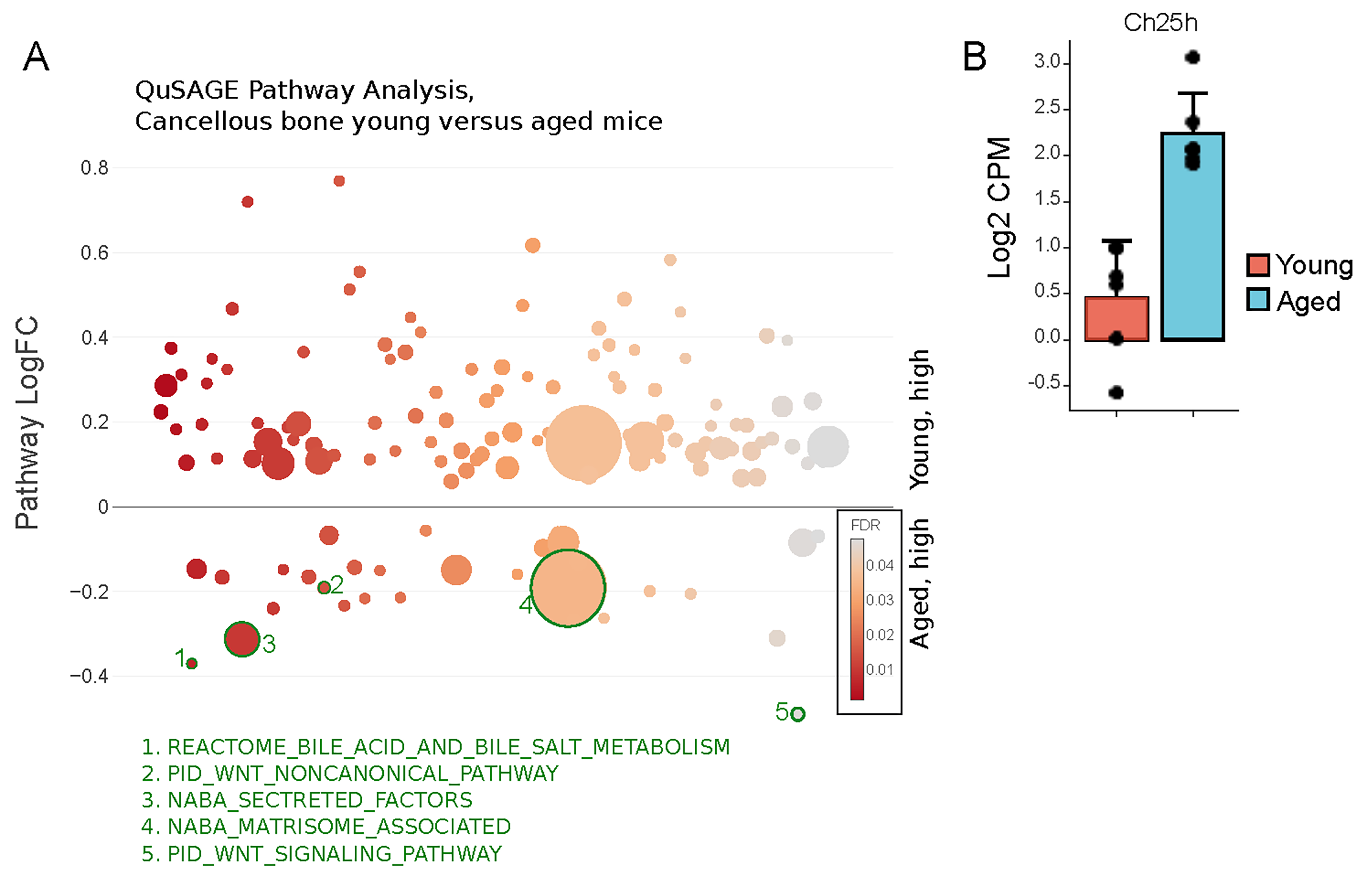

Supplement: Supplementary file 2 — Supplemental Fig. S2. (A) QuSAGE differential pathway analysis in cancellous bone from young and aged mice. All pathways with less than 10 genes were discarded and only pathways with FDR <0.05 are shown. The y axis shows pathway‐wide log‐transformed fold change between young and aged mice and the x axis shows the FDR value. The size of each circle is proportional to the number of genes in a pathway. (B) Differential expression of cholesterol 25‐hydroxylase (Ch25h) between young and old cancellous bone. [file JBM4-5-e10535-s002.tif]

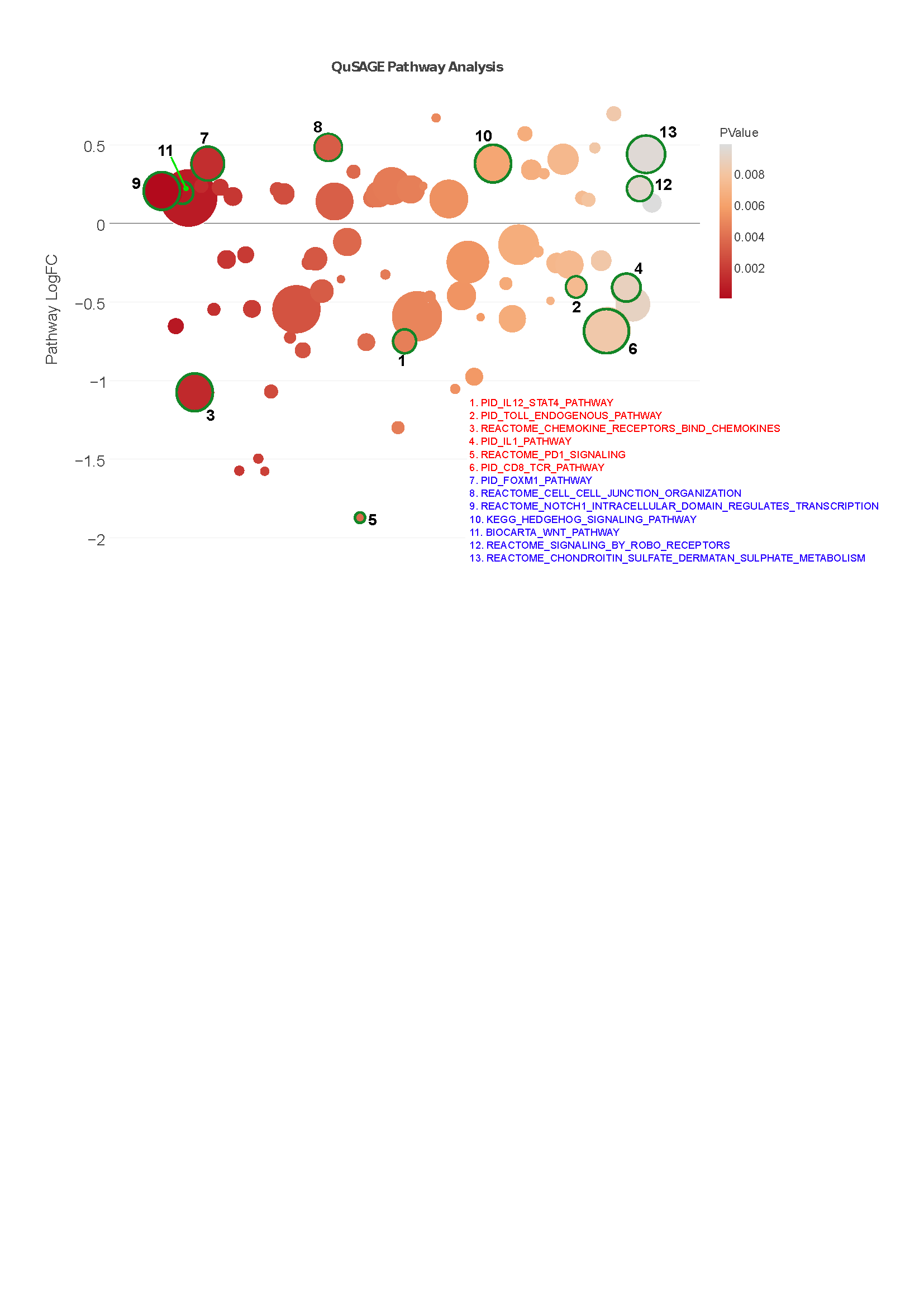

Supplement: Supplementary file 3 — Supplemental Fig. S3. QuSAGE differential pathway analysis of the peri‐implant tissues from young and aged mice. All pathways with less than 10 genes were discarded and only pathways with a p value <0.01 are shown. Other than that low stringency gene expression filtering (group cpm > 0.3) was performed before the analysis; the pathway analysis was performed as described in Materials and Methods and Fig. 2B. The y axis shows pathway‐wide log‐transformed fold change between young and aged mice and the x axis shows the p value. The size of each circle is proportional to the number of genes in a pathway. Pathways that are upregulated in aged mice are shown in red and young mice in blue. [file JBM4-5-e10535-s008.tif]
